# Pediatric Nutrition and Its Role in Preventing Non-communicable Diseases: A Review

**DOI:** 10.7759/cureus.87431

**Published:** 2025-07-07

**Authors:** Aakansha Maria Rajeev, Harshini Malisetty, Omkar Prasad Baidya, Krishna Vamshy J, Shilpi Siddhanta, Binthuja G Dharan

**Affiliations:** 1 Department of Medical Sciences, SDM (Shri Dharmasthala Manjunatheshwara) College of Medical Sciences and Hospital, Dharwad, IND; 2 Department of Medicine, Government Medical College, Mahbubnagar, Mahbubnagar, IND; 3 Department of Physiology, Jagannath Gupta Institute of Medical Sciences and Hospital, Kolkata, IND; 4 Department of Paediatrics, Sri Siddhartha Academy of Higher Education, Tumakuru, IND; 5 Department of Paediatrics, Eastern Railways Hospital, Liluah, Howrah, IND; 6 Department of Paediatrics, B.R. Singh Hospital, Kolkata, IND; 7 Department of Siddha and Yoga Medicine, Government Siddha Medical College - Palayamkottai, Tamil Nadu Dr. M.G.R. Medical University, Tirunelveli, IND

**Keywords:** artificial intelligence, childhood obesity, complementary feeding, nutrigenomics, pediatric nutrition, public health policies, zinc deficiency

## Abstract

Pediatric nutrition is crucial for the prevention of non-communicable diseases (NCDs), which increasingly impact children and adolescents worldwide. Nutritional interventions during early childhood, such as exclusive breastfeeding in the first six months and the introduction of nutrient-rich complementary foods thereafter, lay the foundation for lifelong prevention of chronic diseases. The growing global burden of NCDs, including obesity, cardiovascular diseases, and diabetes, highlights the urgent need for effective public health strategies to promote healthy dietary habits and physical activity among young populations. This review explores the role of nutrition in preventing NCDs, examining key dietary guidelines and recommendations from global organizations such as the Food and Agriculture Organization (FAO), the World Health Organization (WHO), and the United Nations International Children's Emergency Fund (UNICEF). Additionally, it identifies barriers to the effective implementation of nutrition policies, including economic constraints, food insecurity, cultural beliefs, urbanization, and misinformation. The review further explores innovative research directions, including the potential of nutrigenomics, artificial intelligence (AI)-based dietary monitoring, and fortified foods. Integrating nutrition into primary healthcare systems and public health initiatives is essential to addressing the root causes of NCDs in children. In conclusion, addressing these challenges through personalized nutrition, technological interventions, and policy reforms will be key to preventing NCDs and promoting lifelong health in children.

## Introduction and background

Essential nutrients are provided to help children grow properly, develop physically, mature cognitively, and maintain a well-developed immune system during the period of childhood, from infancy to adolescence. This is a significant aspect of public health, as it establishes both immediate health and the long-term physiological resilience of a child [1].

Pediatric nutrition differs from adult dietary considerations in terms of distinctive metabolic needs, rapid developmental changes, and a special vulnerability to imbalances in nutrient supply. Nutritional adequacy in early life stages is critical not only to ensure lifelong health, but also because the nutritional environment lays the groundwork that determines whether a child will develop susceptibility to disease or, conversely, resilience against it [2].

The global problem of non-communicable diseases (NCDs) has risen rapidly in adults, children, and adolescents. NCDs - primarily cardiovascular illnesses, diabetes, cancer, and chronic respiratory problems - are the major cause of death globally, resulting in over 75% of all deaths, with nearly 43 million occurring in 2021 [3]. The traditional view of NCDs as adult-onset illnesses has shifted recently, due to epidemiological trends showing a concerning increase in early markers of NCDs among younger populations. For example, childhood obesity has become an epidemic, and the World Health Organization (WHO) reports that over 37 million children under the age of five were overweight or obese in 2022 [4]. There is now a higher incidence of insulin resistance, hypertension, dyslipidemia, and other metabolic alterations diagnosed in school-aged children and adolescents [5].

This early onset of NCDs is due to multiple interconnected factors, of which poor nutritional habits during the pediatric years are most important. The traditional, wholesome diets that are common in many locales have now been displaced by a global nutritional transition, in which people are eating more energy-dense, nutrient-poor foods that are rich in sugars, trans fats, and sodium [6]. Sedentary lifestyles, urbanization, aggressive advertising of unhealthy food products to children, and socioeconomic inequalities further aggravate the problem. Both forms of malnutrition - under- and over-nutrition - continue to place a double burden on low- and middle-income countries. Insufficient intake of vital vitamins and minerals results in poor physical and mental development, while high caloric intake predisposes children to obesity and associated comorbidities [7].

It is increasingly supported by the scientific literature that nutritional experiences during the first 1000 days of life (from conception until the child’s second birthday) are an influential “programming” factor for future health outcomes [8]. The dependence on both deficits and excesses of dietary intake lies in this developmental window. Various studies provide evidence that parental preconception nutrition, as well as early-life nutrition, influence immune function, endocrine regulation, organogenesis, and metabolic pathways, which, in turn, affect susceptibility to these NCDs in later life [9,10]. Exposure to hypercaloric diets during the prenatal and early childhood stages has been linked with increased adiposity, insulin resistance, and dysregulation of lipid metabolism later in adolescence and adulthood [11]. Exclusive breastfeeding, proper complementary feeding, and an adequate supply of micronutrients have protective effects against obesity, type 2 diabetes mellitus, and risk factors for cardiovascular disease [12].

Pediatric nutrition affects not only the health of the individual but also the public health systems. Worldwide, the economic burden of NCDs is expected to be more than $2 trillion per year, and this is due not only to medical expenses but also to impaired productivity, disability, and lower quality of life [13]. Previous research shows that addressing nutritional risk factors early in life can be done using cost-effective, population-wide strategies for disease prevention. Proven interventions with long-term benefits include school feeding programs, public education campaigns, micronutrient fortification, and regulatory policies aimed at food quality and advertising [14].

The implementation and efficacy of pediatric nutrition programs are still heavily lacking and deficient in under-resourced settings. However, access to nutritious food still varies among communities, caregivers remain unaware of how to feed their children to prevent NCDs, and nutrition in primary healthcare services is still not mainstream [15]. Additionally, there is a requirement for new clinical guidelines and culture-specific nutritional interventions that address undernutrition and overnutrition in various populations [16].

The review aims to critically assess the prevention of NCDs in pediatric nutrition. The objective is to analyze the effect diet has on obesity, diabetes, hypertension, and other metabolic disorders, and to consider the effectiveness of nutritional interventions carried out during childhood. It aims to provide evidence-based suggestions for healthcare professionals, educators, policymakers, and caregivers to endorse lifelong health outcomes, starting early in life, through this analysis.

## Review

Overview of pediatric nutritional requirements

Nutrition of pediatric patients is significant for optimal growth and development and the prevention of disease. It entails ensuring that optimal quantities of macronutrients and micronutrients are present at different stages of childhood for healthy physical and cognitive development [17]. The main energy sources and structural components for children are macronutrients, or proteins, fats, and carbohydrates. Proteins are important for growth and tissue repair, as is the case with children who need approximately 9.1 grams per day, and adolescents who need up to 34 grams per day [18]. Children need 130 grams of carbs daily to provide the necessary fuel for energy and brain function, and fats, which are important for brain development, should make up 25%-35% of total energy intake [19].

Iron, calcium, zinc, and vitamins A and D, along with other essential micronutrients, have important roles in growth, immune function, and development. Infants need 0.27 mg/day of iron, and adolescents, 8-11 mg, depending on sex, but iron is very important to prevent anemia, and it promotes cognitive development [20]. It is known that calcium has a role in bone health and development and that the recommended intake increases from 200 mg/day in infants to 1300 mg/day in adolescents [21]. Vitamin A helps in vision and immune function, and vitamin D absorbs calcium and keeps bones healthy. Prevention of long-term deficiencies is achieved by adequate intake during the early years [22].

Nutrition needs are different at various stages of development. It is recommended to breastfeed consistently for the initial six months of infancy, as it provides critical nutrients and antibodies. In the process of weaning children to solid foods, complementary feeding should contain a variety of foods so that the growing nutritional needs are met. A well-balanced supply of nutrition in childhood and adolescence is the basis for a lifetime of health and helps to limit the risks of NCDs [23]. The age-specific recommendations for dietary nutrition are mentioned in Table 1.

**Table 1:** Age-Specific Recommended Daily Nutrient Intake in Pediatric Populations

Nutrient	0-6 Months (F)	0-6 Months (M)	7-12 Months (F)	7-12 Months (M)	1-3 Years	4-8 Years	9-13 Years (F)	9-13 Years (M)	Reference
Energy (kcal/day)	520	570	676	743	1000	1200-1400	1600-2000	1800-2200	[24]
Protein (g/day)	9.1	9.1	11.0	11.0	13.0	19.0	34.0	34.0	[25]
Fat (g/day or % of kcal)	31 (40-55%)	31 (40-55%)	30 (35-50%)	30 (35-50%)	30-40%	25-35%	25-35%	25-35%	[26]
Carbohydrate (g/day)	60	60	95	95	130	130	130	130	[27]
Iron (mg/day)	0.27	0.27	11.0	11.0	7.0	10.0	8.0	11.0	[28]
Calcium (mg/day)	200	200	260	260	700	1000	1300	1300	[29,30]
Vitamin A (µg/day)	400	400	500	500	300	400	600	600	[31]
Vitamin D (IU/day)	400	400	400	400	600	600	600	600	[32,33]
Zinc (mg/day)	2.0	2.0	2.5	2.5	5.0	5.0	8.0	8.0	[34]
Folate (µg/day)	65	65	80	80	150	200	300	300	[35]

Epidemiology of NCDs in children and adolescents

The prevalence of NCDs in children and adolescents is being increasingly noticed worldwide. The public health concern regarding the rising incidence of obesity, diabetes, hypertension, and metabolic syndrome among the young population is growing. According to estimates by the WHO, childhood obesity has nearly doubled over the last 40 years. This is one of the conditions strongly linked to the early onset of type 2 diabetes, which is rising dramatically in children and adolescents, mainly due to poor eating habits and insufficient physical exercise. Folate deficiency remains a prevalent nutritional concern in many populations, including children and adolescents, where insufficient intake can impair growth, cognitive development, and metabolic health [36]. Metabolic syndrome, characterized by a cluster of risk factors including elevated blood pressure, abnormal cholesterol levels, and insulin resistance, is now increasingly diagnosed in adolescents and often leads to long-term wellness issues such as cardiovascular disease [37].

There are also important regional and socioeconomic differences in the epidemiology of NCDs in children. In high-income countries, obesity and metabolic disorders are more likely to be caused by the lifestyle factors of excessive caloric intake and sedentary behavior. On the contrary, undernutrition is still prevalent in low- and middle-income countries, but the rise of obesity and related diseases, alongside stunting and micronutrient deficiencies, is giving rise to a "double burden" of malnourishment [38].

The emergence of NCDs in children is due to an intricacy of dietary, genetic, and environmental interactions. Rising obesity and diabetes rates are from diets rich in processed food, sugar, and low physical activity [39]. Also, some children have a genetic predisposition to obesity and insulin resistance, especially when combined with poor environmental factors. In addition, urbanization, changes in food marketing, and a decline in physical activity opportunities in many areas have added to the problem [40]. Consequently, NCD prevention in children must be multifactorial and include modification of dietary habits and physical activity, as well as addressing environmental factors, and accounting for genetic susceptibility.

Mechanistic link between nutrition and NCDs

Poor nutrition has a complex relationship to the development of NCDs. One of the fundamental metabolic disturbances that can lead to several NCDs, such as type 2 diabetes and cardiovascular diseases, is insulin resistance, which is typically the result of a high-glycemic diet [41]. Refined carbohydrates and sugars in excess cause a rapid rise in blood glucose, which, in turn, forces the pancreas to secrete an excess of insulin. Eventually, this hyper-insulinemic state leads to insulin resistance, meaning the cells in the body become less responsive to insulin, further fueling metabolic dysfunction. Elevated blood glucose levels, dyslipidemia, and enhanced fat accumulation - especially in the abdominal region - are all important peril factors for diabetes, obesity, and heart disease, and are also involved in this process [42].

The role of poor dietary habits in the development of NCDs is also related to inflammatory pathways that are triggered by these habits. The body’s inflammatory response is triggered by diets that are high in processed foods, trans fats, and sugars. The inflammatory state is associated with the pathogenesis of several NCDs, i.e., type 2 diabetes, atherosclerosis, and some cancers. Visceral fat, which develops as a result of consuming high-glycemic diets, secretes pro-inflammatory cytokines that predispose to insulin resistance and endothelial dysfunction, and lead to cardiovascular disease [43].

Also, a dysregulated gut microbiome results from poor dietary patterns, such as those high in processed carbohydrates and fats, and leads to chronic inflammation, resistance to insulin, and metabolic dysfunction. The gut bacteria can modulate immune responses, influence the metabolism of food, and even affect the axis of the gut and brain. Hence, diet plays a role in chronic disease susceptibility [44]. The mechanisms linking diet and insulin resistance are mentioned in Table 2.

**Table 2:** Mechanisms Linking High-Glycemic Diets and Insulin Resistance in the Development of NCDs NCD, Non-communicable Disease; NAFLD, Non-alcoholic Fatty Liver Disease

Mechanism	High-Glycemic Diets	Insulin Resistance	Impact on Metabolism	Associated NCDs	Reference
Increased Blood Glucose Levels	Rapid rise in glucose levels after meals	Leads to the overproduction of insulin by the pancreas	Chronic elevation in blood sugar levels	Type 2 Diabetes, Cardiovascular Diseases	[45]
Insulin Hypersecretion	Due to high carbohydrate intake	Pancreatic β-cells overcompensate by producing excess insulin	Over time, the pancreas exhausts its ability to secrete insulin efficiently	Metabolic Syndrome, Obesity	[46]
Increased Fat Storage	Elevated insulin promotes fat storage	Insulin resistance leads to adipocyte dysfunction	Accumulation of visceral fat	Type 2 Diabetes, NAFLD	[47]
Reduced Lipid Oxidation	High insulin inhibits lipid oxidation	Decreased ability to burn stored fat	Increased fat deposition and weight gain	Obesity, Cardiovascular Disease	[48]
Chronic Inflammation	Poor diet triggers immune activation	Insulin resistance induces low-grade systemic inflammation	Inflammation of adipose tissue, liver, and vascular system	Type 2 Diabetes, Atherosclerosis	[49]
Increased Visceral Fat	High-glycemic foods increase fat accumulation in the abdominal region	Abdominal fat is particularly sensitive to insulin resistance	Larger fat deposits lead to further metabolic disturbances	Hypertension, Stroke, Cardiovascular Diseases	[50]
Dysregulation of Hormonal Signals	High sugar intake alters leptin, ghrelin signaling	Impairment in satiety hormones increases hunger	Disrupts appetite regulation, leading to overeating	Obesity, Type 2 Diabetes	[51]

Dietary patterns and interventions

Dietary patterns and interventions are effective means for the prevention and management of NCDs. Very well-established dietary approaches with great health benefits, including reducing the risk of chronic disease, include the Mediterranean diet, plant-based diets, and the Dietary Approaches to Stop Hypertension (DASH) diet. A Mediterranean diet high in fruits, vegetables, whole grains, olive oil, and moderate intakes of fish is associated with a decrease in cardiovascular diseases, diabetes, and some cancers [52]. This diet focuses on healthy fats, fiber, and antioxidants, which are important in reducing inflammation and improving lipid profiles - both of which are key factors in NCD prevention [53].

**Figure 1:**
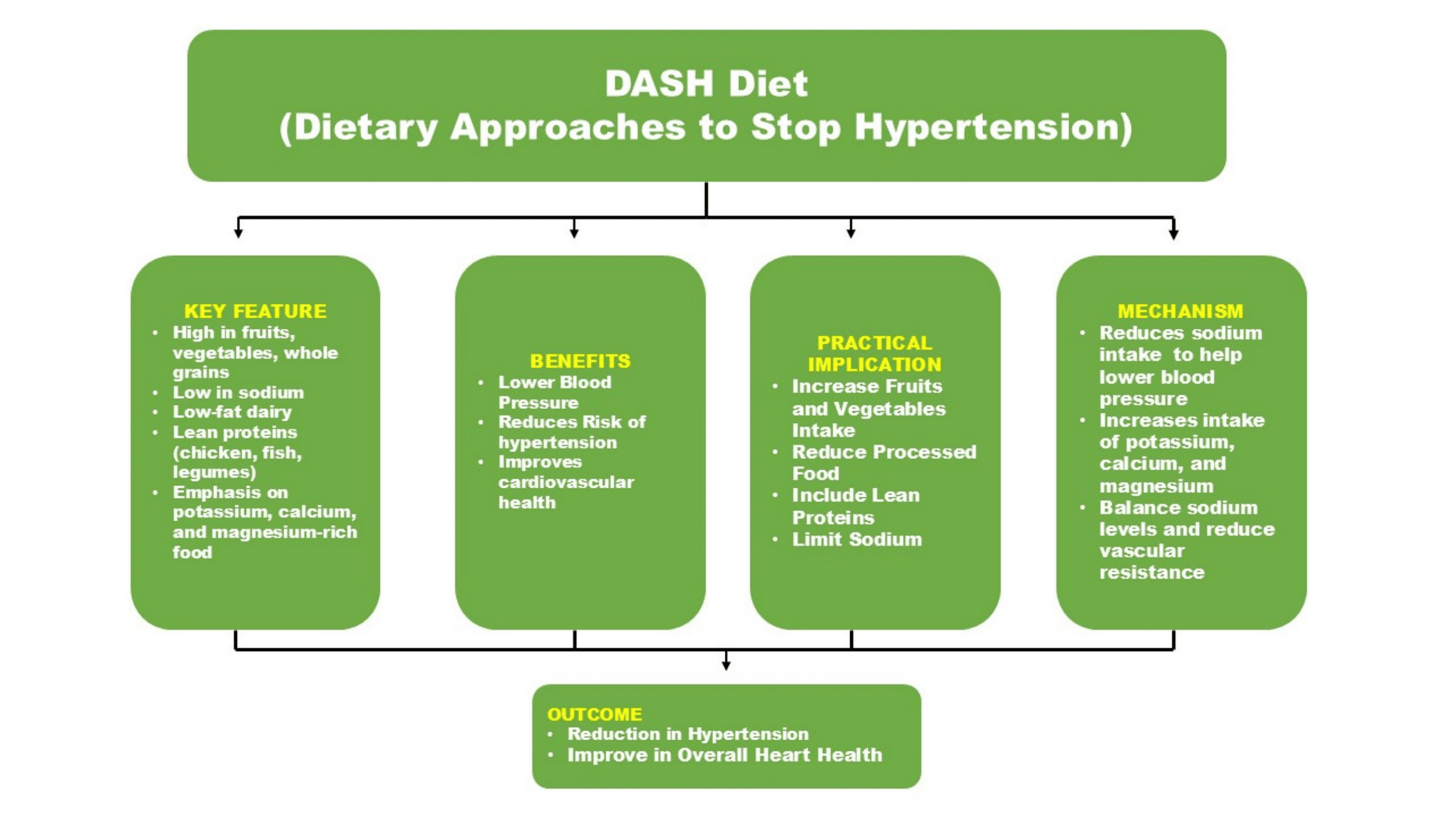
DASH Diet and Its Role in Reducing Hypertension Image Credit: Aakansha Maria Rajeev DASH, Dietary Approaches to Stop Hypertension

These diets are rich in vegetables, fruits, legumes, nuts, and whole grains, providing high levels of fiber, vitamins, and minerals while being low in unhealthy fats and cholesterol [54]. Plant-based diets have propagated evidence of improving metabolic health, reducing oxidative stress, and lowering blood pressure, which are important to manage and prevent NCDs [55]. The DASH diet, which is designed particularly to lower blood pressure, aims to reduce sodium intake while increasing potassium-rich foods like fruits, vegetables, and low-fat dairy products. It has been shown in studies that the DASH diet decreases systolic and diastolic blood pressure and improves cardiovascular health overall [56]. The DASH diet features and its role in reducing hypertension are mentioned in Figure 1.

Apart from individual dietary patterns, school-based and community-based nutritional interventions are crucial in addressing NCDs at the population level. The objective of these interventions is to enhance dietary habits, boost physical activity, and create a healthier environment for children and adolescents. It is proven that school nutrition programs, such as offering healthier meal options and nutrition education, reduce rates of childhood obesity and promote the health of students when implemented [57]. Public health campaigns at the community level and support for healthier food environments promote long-term behavior changes.

Policy, guidelines, and global strategies

NCDs constitute a major global burden, and therefore, international organizations such as the WHO, the United Nations International Children's Emergency Fund (UNICEF), and the Food and Agriculture Organization (FAO) have developed comprehensive guidelines to improve nutrition and prevent diet-related diseases. A balanced diet, regular physical activity, and a reduction in salt, sugar, and unhealthy fats are promoted by the WHO’s Global Strategy on Diet, Physical Activity, and Health plan [58]. The plan encourages people to eat high amounts of fruits, vegetables, whole grains, lean protein, and low-fat dairy, and to limit quantities of processed foods and sugary beverages.

As part of the Infant and Young Child Feeding (IYCF) guidelines, UNICEF focuses on the early nutrition of children. UNICEF supports exclusive breastfeeding for the first six months, followed by complementary feeding with nutrient-dense meals, such as vegetables, fruits, and fortified cereals, which help reduce malnutrition and prevent chronic diseases later in life [59].

FAO has a vital role to play in global nutrition advocacy for diversified diets and food fortification in reducing malnutrition. Increasing the intake of plant-based foods, i.e., legumes, nuts, and whole grains, can be suggested in order to prevent nutrient deficiencies and associated disease states, like anemia and osteoporosis [60].

Programs at the national level, like Integrated Child Development Services (ICDS) by India and MyPlate in the US, develop recommendations specific to nutrition at the national level, aiming at self-assessed balanced meals and food security for the most vulnerable [61].

**Figure 2:**
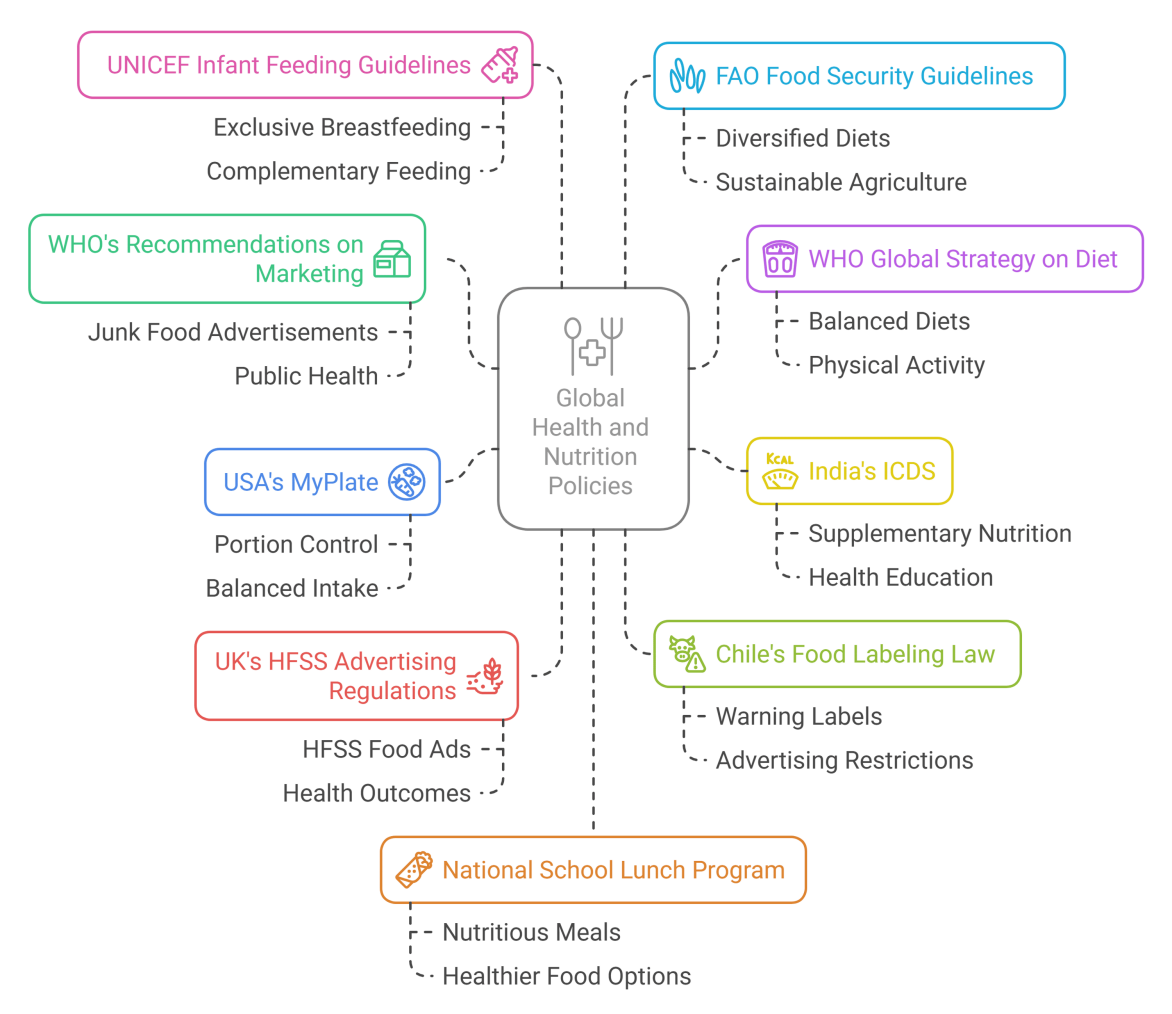
Global Strategies and Policy Recommendations for Nutrition Image Credit: Aakansha Maria Rajeev UNICEF, United Nations International Children's Emergency Fund; FAO, Food and Agriculture Organization; WHO, World Health Organization; ICDS, Integrated Child Development Services; HFSS, High Fat, Salt, or Sugar

The recommended policies set by the WHO for junk food marketing are aimed at reducing the exposure of children to advertisements that promote unhealthy foods. The Chilean pioneering law, which prohibits junk food advertising during children's programs and mandates clear warning labels for unhealthy products, has resulted in a strong reduction in children's consumption of unhealthy foods [62]. The strategies and policy recommendations are illustrated in Figure 2.

Barriers and challenges

There are economic, cultural, urbanization, and digital media barriers to addressing NCDs through improved nutrition. Low- and middle-income nations are facing the most pressing challenges, which include economic constraints. People living in low-income families tend to be unable to afford healthy, nutritious foods and instead rely on more available and cheaper energy-dense, low-nutrient options [63]. These dietary patterns are associated with higher rates of obesity, diabetes, and other chronic diseases [40,62]. Food insecurity exacerbates malnutrition, particularly among vulnerable individuals like children and elders, intensifying the global NCD burden. National nutrition programs intended for low-income communities are also not effective as a result of economic instability and rising food prices [64].

There are cultural beliefs and misinformation about nutrition itself, making it difficult to influence dietary habits. Traditional dietary practices may be deeply ingrained and thus resist change, especially if high-fat or high-sugar foods have been culturally valued in a society. When people receive misinformation on social media or from unverified sources, they may practice harmful diets, which undermine public health messaging [65].

The fast rate of urbanization has also transformed dietary habits as people relocate from rural to urban areas and adopt Westernized diets high in fats, sugars, and refined carbohydrates [66]. This shift - characterized by high energy intake and reduced physical activity - has contributed to the increase in obesity, hypertension, and type 2 diabetes. There is limited access to fresh produce in urban environments, which complicates efforts to implement successful nutrition interventions [67].

Future perspectives and research directions

Innovation in the use of technology, personalized strategies, and systemic integration represents the future of pediatric nutrition and the prevention of NCDs. The science of nutrigenomics, which explores how genes interact with diet, may represent an important means by which nutrition can be tailored to fit the genetic makeup of the individual [68]. Understanding genes and how certain nutrients modulate metabolism enables future dietary interventions that are best suited for each child and address problems such as obesity, metabolic syndrome, and many other NCDs. Traditionally, personalized pediatric diets based on nutrigenomic research are expected to provide more precise and effective dietary recommendations and improve health outcomes [68].

Artificial intelligence (AI) and digital tools are being used to change the way people monitor and control their diets. With the help of AI, apps and wearable devices can monitor dietary intake and give real-time, personalized feedback, which can make interventions more efficient. With these tools, healthcare providers can measure the impact of dietary changes remotely, and this is valuable information that can inform eating behavior modification. These technologies can be used to analyze large-scale population data and predict the long-term impact of various dietary patterns on health outcomes [69].

Another promising approach to address deficiencies in micronutrients is the fortification of staple foods with essential nutrients in low-resource settings. Deficiencies of iron, vitamin A, iodine, and zinc are key contributors to many nutrition-related diseases, and these can be reduced with biofortified crops and improved or enhanced supplements [70]. Finally, nutrition should be integrated into primary care and public health systems to prevent NCDs. Through the integration of nutrition into regular healthcare, healthy living can be promoted from an early age, and all populations can be given the dietary assistance required [71].

## Conclusions

Pediatric nutrition is important, as it enhances lifelong health and minimizes the chances of NCDs at a tender age. Childhood interventions, including exclusive breastfeeding, balanced complementary feeding, and the right amount of the necessary nutrients, can greatly decrease the chances of developing conditions such as obesity, diabetes, and cardiovascular disorders. As the number of these diseases in young people is increasing, there is an urgent need to have effective public health measures that promote healthy eating and physical activity at the youngest age of life. Even though there has been improvement, there are still numerous factors that limit the success of nutritional programs, such as food insecurity, socioeconomic inequality, and misinformation. In the future, solutions to the problem of pediatric health outcomes will be possible through personalized nutrition plans, technology-based dietary surveillance, and food fortification. A key to long-term and sustainable benefits of nutritional support to children in different settings will be to strengthen the process of integration of nutritional support in primary healthcare, communities, and systems.
